# Anxiety about anxiety: a survey of emergency department provider beliefs and practices regarding anxiety-associated low risk chest pain

**DOI:** 10.1186/s12873-018-0161-x

**Published:** 2018-03-14

**Authors:** Paul I. Musey, John A. Lee, Cassandra A. Hall, Jeffrey A. Kline

**Affiliations:** 1 Department of Emergency Medicine, Indiana University School of Medicine, Indianapolis, IN 46202 USA; 2 University of Virginia School of Medicine, Charlottesville, Virginia, 22908 USA

**Keywords:** Emergency department, Chest pain, Anxiety, Psychological conditions

## Abstract

**Background:**

Approximately 80% of patients presenting to emergency departments (ED) with chest pain do not have any true cardiopulmonary emergency such as acute coronary syndrome (ACS). However, psychological contributors such as anxiety are thought to be present in up to 58%, but often remain undiagnosed leading to chronic chest pain and ED recidivism.

**Methods:**

To evaluate ED provider beliefs and their usual practices regarding the approach and disposition of patients with low risk chest pain associated with anxiety, we constructed a 22-item survey using a modified Delphi technique. The survey was administered to a convenience sample of ED providers attending the 2016 American College of Emergency Physicians Scientific Assembly in Las Vegas.

**Results:**

Surveys were completed by 409 emergency medicine providers from 46 states and 7 countries with a wide range of years of experience and primary practice environment (academic versus community centers). Respondents estimated that 30% of patients presenting to the ED with chest pain thought to be low risk for ACS have anxiety or panic as the primary cause but they directly communicate this belief to only 42% of these patients and provide discharge instructions to 48%. Only 39% of respondents reported adequate hospital resources to ensure follow-up. Community-based providers reported more adequate follow-up for these patients than their academic center colleagues (46% vs. 34%; *p* = 0.015). Most providers (82%) indicated that they wanted to have referral resources available to a specific clinic for further outpatient evaluation.

**Conclusion:**

Emergency Department providers believe approximately 30% of patients seeking emergency care for chest pain at low risk for ACS have anxiety as a primary problem, yet fewer than half discuss this concern or provide information to help the patient manage anxiety. This highlights an opportunity for patient centered communication.

**Electronic supplementary material:**

The online version of this article (10.1186/s12873-018-0161-x) contains supplementary material, which is available to authorized users.

## Background

Chest pain is one of the most common chief complaints evaluated in the Emergency Department (ED), accounting for approximately 20% of all annual ED visits nationwide [1]. A small minority of these patients have a true cardiopulmonary emergency such as acute coronary syndrome (ACS) or pulmonary embolism (PE). Frequently, these patients have psychosomatic contributors to their symptoms, including panic or anxiety disorders. These disorders are present in up to 58% [2] of patients presenting with chest pain but remain undiagnosed in approximately 80% of these patients [3]. Many adults with chest pain undergo extensive ED evaluations to rule out cardiopulmonary emergencies. In some cases, these evaluations can last 48 h including multiple tests, radiation exposure, and high cost, only to yield no named diagnosis [4]. A significant fraction of these patients go on to develop chronic chest pain and continue to seek medical attention despite negative cardiac evaluations [2]. Prior work has indicated emergency providers are hesitant to diagnose and discuss the role of anxiety [4] as a significant co-factor or etiology of their pain. However, the beliefs of ED providers regarding the prevalence of anxiety among patients with low risk chest pain and how they manage those patients both in the ED and at discharge have not been evaluated together.

### Objective

We sought to evaluate ED provider beliefs regarding patients with low risk chest pain thought to be secondary to anxiety symptoms. Further, we wanted to evaluate if there was a discrepancy between these beliefs and their management practices.

## Methods

This work was a hypothesis generating survey, conducted in accordance with guidelines by Mello et al., including both expert consensus and a modified Delphi technique [5, 6]. This protocol was deemed to be exempt by the IRB (protocol# 1601415405) at Indiana University School of Medicine. Authors PIM, JAL, CLH, and JAK initially determined that the objective of the hypothesis generating survey was to: evaluate the gap between ED provider beliefs regarding the approach and disposition of patients with low risk chest pain thought to be secondary to anxiety compared with their perceived practice patterns. The authors developed an initial set of themes which led to the generation of a 15-item survey which was test-administered to ED providers at Indiana University Department of Emergency Medicine. Responses from this test survey were analyzed along with feedback from participants by an advisory group who determined the final themes, domains, and survey architecture via a modified Delphi technique. This advisory group included individuals with expertise in both the subject areas as well as survey generation and administration in order to ensure face, content, and construct validity. The authors then generated a draft survey consisting of 26 items which were again vetted by the advisory group. Through an iterative process and consensus, edits were made and the items were reduced to a final survey consisting of 22 items. These included a mixture of multiple choice and visual analogue scale (0–100%) questions (Additional file 1: Appendix 1).

The survey was then administered to a convenience sample of Emergency Medicine (EM) providers visiting the exhibitor hall at the 2016 American College of Emergency Physicians Scientific Assembly (ACEP16) in Las Vegas, Nevada from October 16–19, 2016. This forum was chosen because it represents the largest annual gathering of EM providers [7]. Eligible survey participants included advanced practitioners (i.e.: nurse practitioners and physician’s assistants), emergency medicine residents, as well as physicians at the fellow or attending level who were practicing in academic and/or community settings. The investigators obtained a booth in the ACEP16 exhibitor hall and invited all eligible participants passing by the vicinity of the booth to complete the survey. A description and invitation to the booth was printed in the ACEP16 program and the meeting’s website. Additionally, the investigators handed out invitation cards at the meeting. To encourage participation, we provided a material incentive in the form of a raffle for a FitBit® activity monitor. The number of passers-by who were approached but declined participation was not tracked.

Participants first viewed a description and purpose of the survey prior to starting, and then completed the survey using either a laptop computer or electronic tablet. Study data were collected and managed using REDCap electronic data capture tools hosted at Indiana University [8]. Exported data were analyzed using Microsoft Excel for Mac, Version 15.14, and IBM SPSS Statistics for Mac, Version 22.0. The survey data was exported from REDCap into both software packages, and both univariate and multivariate analysis was completed. Where appropriate, 95% confidence intervals are reported. All visual analog-scale data were found to be non-normally distributed by both Kolmogorov-Smirnov and Shapiro-Wilk normality testing. All data generated or analyzed during this study are included in this published article as a supplemental excel dataset (The complete dataset generated from this survey and the basis for this manuscript is available from the authors upon request).

## Results

**Fig. 1:**
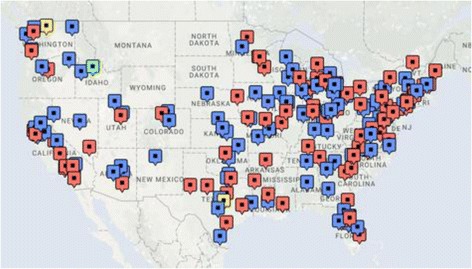
Map of Providers by Practice Type and Location. Map of the continental United States with flags denoting the practice location and type of practice (academic, community, urgent care, or other) KEY: Red – Academic Practice, Blue – Community Practice, Green – Urgent Care Practice, Yellow – Other *Note: 2 responses from Alaska and 2 responses from Hawaii. An additional 29 responses were international (14 from Canada)

Four-hundred-and-nine surveys were completed, representing approximately 5% of ACEP16 conference attendees. There was diversity in respondent geographic location (Fig. 1), years of experience, and primary practice environment. The majority of respondents practiced at academic centers (52%), had less than 10 years of experience (68%), and were male (72%) as shown in Table 1. Twenty-nine respondents (7.0%) identified their primary practice location as outside of the United States.

**Table 1:** Provider gender, practice environment, and level of experience

	Male	Female	Other/No Response	
Practice Environment				
Academic	151	63	0	214 (52%)
Community	131	45	0	176 (43%)
Urgent/Other	12	5	2	19 (5%)
				409 (100%)
Experience				
Advanced Practitioner	12	12	1	25 (6%)
Current Resident	68	28	0	96 (24%)
Fellow/Attending 0-4 yrs	52	32	0	84 (20%)
Attending 5-9 yrs	53	20	0	73 (18%)
Attending 10 + yrs	109	21	1	131 (32%)
	294 (72%)	113 (28%)	2	409 (100%)

### Current practice patterns

**Fig. 2:**
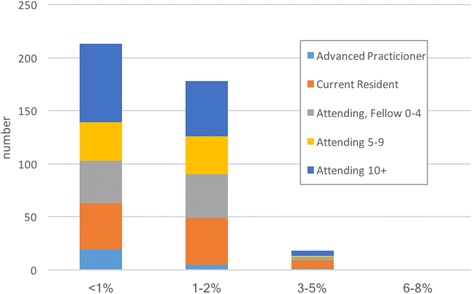
Acceptable ACS Miss Rate by Experience. Stacked bar graph depicting participants selection of what they deem to be an acceptable ACS miss rate. Each colored stack in the bar corresponds to the provider position and/or experience level as noted on the graph

A majority of providers believed an “acceptable ACS miss rate” was either < 1% (52%) or 1–2% (44%) with only 4% of respondents willing to accept an ACS miss rate of 3–5% illustrated in Fig. 2. Providers estimated that 30% (95%CI 28–32) of patients presenting to the ED with chest pain, which they have stratified to be low risk by whatever method, have anxiety or panic as the primary cause as shown in Table 2. Of those patients, the majority are female (38% male, 95%CI 36–39). For patients whom they believe anxiety or panic is the primary problem, respondents indicated that they directly communicate this belief to only 42% of these patients, and offer anxiolytic treatment to only 41% in the ED. Additionally, they offer discharge instructions and prescriptions for anxiolytics in 48 and 21%, respectively. Though 54% of respondents indicated they believe they have a professional responsibility to provide patients with an actual ICD-code diagnosis of anxiety when life threats are ruled out, providers report documenting a specific ICD-code diagnosis of “anxiety” or “panic” only 29% of the time (95%CI 27–32). Providers appeared more likely to diagnose anxiety in patients under age 25 (Additional file 2: Table S1).

**Table 2:** Attitudes and practices for patients with chest pain who are risk stratified as low risk for ACS (by whatever method)

	Mean (95% CI)		Mean (95% CI)
What % have anxiety or panic as primary cause of their symptoms?	30 (28–32)	How often do you provide anxiety/panic specific treatment in the ED for these patients?	41 (38–43)
Of these patients, what % are male?	38 (36–39)	How often do you provide any anxiety/panic specific prescriptions for these patients?	21 (19–24)
How often do you specifically tell these patients that you believe anxiety or panic may be the causing their symptoms?	42 (39–46)	How often do you discharge these patients with information or instructions about anxiety or panic?	48 (45–51)
		How often do you discharge these patients with and ICD diagnosis of “anxiety” or “panic”?	29 (27–32)

### Addressing the problem of anxiety

Thirty-nine percent (161/407) of respondents reported adequate hospital resources to ensure follow-up for patients with low risk chest pain they suspect is secondary to anxiety. Community-based providers reported more adequate follow-up for these patients than their academic center colleagues (46% of community providers responded that they have adequate follow-up available, versus only 34% of academic center providers; 2-sided Fisher’s exact test 0.015), (Table 3). When asked about possible strategies that could be used to improve management for patients with chest pain due to anxiety, most providers (82%) said that it would helpful to have a referral available to a specific clinic for further outpatient evaluation. In discussing what tools would make a provider more comfortable diagnosing and referring these patients (Table 4), practice patterns of colleagues (48%, 95%CI 45–50) and local hospital policy (56%, 95%CI 53–59) were found to be less helpful than a multicenter trial (74%, 95%CI 72–76) or professional organization practice guidelines (71%, 95%CI 69–73).

**Table 3:** Perceived ability to provide adequate follow-up

Do you believe you have adequate resources to ensure appropriate outpatient follow up for these patients?	# of Academic Providers [% of total]	# of Community/Urgent Care/ Other Providers [% of total]	Total
Yes	72 [17.6%]	89 [21.7%]	161
No/Unsure/No Response (Total)	113/28/1 (142) [34.7%]	82/23/1 (106) [25.9%]	248
Total	214	195	409

Our Fisher's Exact test revealed that the proportion of providers who do not believe or are unsure if they have adequate resources significantly differed by type of practice (academic vs. community), *p* = 0.015

**Table 4:** Tools necessary to increase provider comfort with diagnosis

To what degree would each of the following increase your level of comfort in making a diagnosis of anxiety in patients with chest pain and providing a referral for treatment?	
	mean (95% CI)
Practice patterns of colleagues	48% (45–50)
Local hospital policy adoption	56% (53–59)
Multicenter trial	74% (72–76)
Professional organization practice guidelines	71% (69–73)

## Discussion

This study incorporated both a diverse geographical representation, and a wide range of practice settings and experience. The main message of this work is that physicians believe that approximately 30% of patients seeking emergency care for low risk chest pain have anxiety or panic as a primary problem, yet fewer than half provide any treatment or information to help the patient manage anxiety. The authors recognize that the primary imperative of emergency care is to protect and intervene against threats to a patient’s life. However, in patients without a serious cardiopulmonary disease, untreated anxiety can degrade quality of life and worsen perceptions of wellness [9, 10], contributing to systemic inflammation [11], which ironically creates the pathophysiology of coronary artery disease [12, 13]. Additionally, this is associated with an increased health resource burden [14–18]. We would argue that the most important of these to the emergency clinician is the detrimental effect on patient quality of life and unnecessary ED resource utilization and recidivism. Prior work in a large cohort of ED patients with chest pain discovered that only 0.2% were given an ICD9 diagnosis of anxiety; however, none of the 8% of patients who self-identified anxiety as likely the cause for their chest pain symptoms at follow-up were given this diagnosis even secondarily [4]. This low rate of anxiety diagnoses in patients with non-life threatening chest pain in the ED was further demonstrated in a national sample using the National Hospital Ambulatory Medical Care Survey, where only 2.3% received this diagnosis [19]. Whereas, a systematic review by Webster et al. incorporated nine studies and found the likely prevalence of diagnosable anxiety in patients with non-cardiac chest pain to be between 21 and 58% [2]. Our current work is consistent with that of Al-Ani et al., who found that 30% of chest pain patients who had been risk-stratified to be low-risk for ACS were identified as suffering from anxiety and 80% of those patients were untreated [20]. This highlights the fact that at least in this evaluation, EM provider gestalt regarding anxiety in the presence of low-risk anxiety is in line with objective measures shown previously. Taken together, these data indicate the need for a more patient-centered approach to communicating with and managing anxiety in patients with low risk chest pain. Helpful methods could include using formal anxiety risk stratification tools such as the Hospital Anxiety Depression Scale (HADS) or Generalized Anxiety Disorder scale (GAD-7). Both of these validated tools have been used to evaluate for the presence of anxiety in this patient population previously [21–23].

Furthermore, even after workup and provider reassurance, patients who present with possible cardiac symptoms are often left with “residual anxiety” despite normal test results [24]. Reassurance of negative test results alone may not help reduce patient concerns about the potentially life-threatening causes of their chest pain [24–26]. Ackerman et al. defined the ideal content for ED discharge communication for patients with chest pain including directed follow-up suggestions and advice on self-care, [24] which are all important parts of the patient-provider interaction, particularly in the emergency department. Our data suggest these steps to be absent in current emergency care and may represent a missed opportunity to affect the trajectory of care [10]. This low rate of discharge communication is likely heavily influenced by the fact that the majority of respondents (61%) found their hospital resources for appropriate follow-up to be inadequate. Not surprisingly, 82% of providers wanted a specific clinic to direct their patients for further evaluation for anxiety.

Implications of these data are that patients could benefit from targeted interventions which minimize psychological distress, improve quality of life [9] and prevent futile emergency department visitation. There are a number of psychological interventions which are available for this patient population which include cognitive behavioral therapy (CBT), relaxation therapy, hyperventilation, hypnotherapy etc. [15]. Foremost and most well established is cognitive behavioral therapy (CBT), which has been shown to be both acceptable to patients and effective in reducing chest pain and improving quality of life [15, 27]. Even brief interventions have been successful as van Beek et al. randomized subjects with non-cardiac chest pain and anxiety after psychological testing to usual treatment vs an abbreviated CBT course (6 sessions) showing a significant decrease in both depression and anxiety symptoms [28]. Unfortunately, CBT interventions are not a readily available resource for ED referral and can be expensive [29]. However, it appears the intervention need not be extensive or expensive, as simply providing self-help information explaining the connection between anxiety and chest pain can make a difference in both psychological and/or physical symptoms [18, 30, 31]. Other anxiety management strategies include training in breathing techniques and mindfulness-based exercises may show effectiveness in this patient population [32–35]. However, any intervention requires a frank conversation about anxiety and appropriate discharge instructions.

### Limitations

The primary limitation of the work is that persons who completed our survey were self- selected by their decision to attend the national meeting, and their willingness to complete our survey. Additionally, we did not provide a formal definition or diagnostic criteria for anxiety to the provider as part of the survey. Thus, these results represent provider beliefs and gestalt based in large part on subjective assessments without the benefit of screening tests such as the HADS [21] or GAD-7 [22, 23] or formal evaluation using the Structured Clinical Interview for DSM Disorders (SCID) [36]. Thus, the objective reality of those beliefs were not assessed or quantified. However, we thought it important to explore as physician gestalt would likely be a trigger for further screening or referral for formal evaluation. Additionally, these screening tools and assessments are not regularly employed in this ED patient population and thus providers may not have been familiar.

## Conclusions

ED providers believe 30% of patients seeking emergency care for chest pain stratified to be low risk for ACS have anxiety as a primary problem, yet fewer than half discuss this concern or provide information to help the patient manage anxiety. These data support the need for a more patient-centered approach to communication and management of anxiety in patients with low risk chest pain. Further investigation to elicit reasons why providers would be hesitant to discuss suspected anxiety in the setting of low risk chest pain and contributing biases is needed.

## Additional files

Additional file 1: Appendix 1. Survey Tool. Survey questions administered to participants. (PDF 60 kb)

Additional file 2. **Table S1.** Hypothetical Patient Scenario. Responses to questions regarding a hypothetical presentation of a patient with chest pain and suspected anxiety or panic. (DOC 224 kb)

## Abbreviation

ACEP16: 2016 American College of Emergency Physicians Scientific Assembly
ACS: Acute coronary syndrome
CBT: Cognitive behavior therapy
ED: Emergency department
EM: Emergency medicine
GAD-7: Generalized Anxiety Disorder – 7
HADS: Hospital Anxiety Depression Scale
PE: Pulmonary embolism
SCID: Structured Clinical Interview for DSM Disorders
