# Supplementary material for: Anxiety about anxiety: a survey of emergency department provider beliefs and practices regarding anxiety-associated low risk chest pain
Source: BMC Emerg Med. 2018 Mar 14;18:10. doi: 10.1186/s12873-018-0161-x (PMC5853064; doi:10.1186/s12873-018-0161-x)
Supplement: Supplementary file 1 — Appendix 1. Survey Tool. Survey questions administered to participants. (PDF 60 kb) [file 12873_2018_161_MOESM1_ESM.pdf]

ACEP 2016 Survey: Anxiety and Non-Cardiac Chest Pain

You are being asked to participate in a survey research project entitled "ED Provider Beliefs and Opinions Regarding Anxiety Associated Non-Cardiac Chest Pain"; which is being conducted by Paul Musey, a faculty member at Indiana University. This survey is anonymous. No one, including the researcher, will be able to associate your responses with your identity. Your participation is voluntary. You may choose not to take the survey or to stop responding at any time. Your completion of the survey serves as your voluntary agreement to participate in this research project.

Thank you!

We are interested in looking at provider beliefs with regards to the evaluation of anxiety or panic in the setting of low risk chest pain in the ED.

The following survey should only take 2-3 minutes to complete and will very helpful in guiding future research pertinent to Emergency Clinicians.

Thank you for your valuable time and input.

1. What is you highest Level of Training
   - ◯ Advanced Practitioner (i.e.: NP, PA)
   - ◯ Current Resident (PGY 1-4)
   - ◯ Staff physician or fellow with 0-4 years in practice
   - ◯ Staff physician with 5-9 years in practice
   - ◯ Staff physician with ≥10 years in practice

2. What is your main practice environment (where you spend >50% of your clinical time)?
   - ◯ Academic center
   - ◯ Community ED
   - ◯ Urgent Care
   - ◯ Other

3. What City, State do you practice in? ______________________________

4. What gender do you identify with?
   - ◯ Male
   - ◯ Female
   - ◯ Other (i.e.: bi-gender, trans-gender, a-gender, etc)

---

5. When you have a patient with chest pain, what do you consider an acceptable miss rate for acute coronary syndrome (ACS)?
   - ◯ a. < 1%
   - ◯ b. 1-2%
   - ◯ c. 3-5%
   - ◯ d. 6-8%

Each of the following questions (#6-14) pertain to patients that you have risk stratified to be low risk for ACS (by whatever method).

Please make a mark on the following visual analog scales to mark your responses.

6. What percentage of these patients do you believe have anxiety or panic as the primary cause for their symptoms?

0% 50% 100%

(Place a mark on the scale above)

7. Of these patients in question 6, what percentage do you estimate are male?

0% 50% 100%

(Place a mark on the scale above)

8. Of these patients in question 6, how often do you specifically tell these patients that you believe anxiety or panic may be causing their symptoms?

Never All the time

(Place a mark on the scale above)

9. How often do you provide any anxiety/panic specific treatment in the ED for these patients?

Never All the time

(Place a mark on the scale above)

10. How often do you provide any anxiety/panic specific prescriptions for these patients?

Never All the time

(Place a mark on the scale above)

11. How often do you discharge these patients with any information or instructions about anxiety or panic?

Never All the time

(Place a mark on the scale above)

12. How often do you specifically discharge these patients with an ICD diagnosis of "anxiety", "panic"?

Never All the time

(Place a mark on the scale above)

13. As a provider, do believe you have adequate hospital resources to ensure appropriate outpatient follow up for these patients?

◯ Yes
◯ No
◯ Unsure

14. Would you find it helpful to have a specific provider/clinic to direct these patients to for outpatient evaluation of anxiety or panic causing their chest pain symptoms?

◯ Yes
◯ No
◯ Unsure

15. For the next three questions consider a patient with chest pain which you suspect is driven by panic or anxiety. This is a patient whom you plan to discharge home, with an age < 50 years, a normal or unchanged ECG, a normal chest x-ray, and a normal troponin.

15a. In this patient how comfortable are you making the diagnosis of anxiety?

Not comfortable at all — Very comfortable

(Place a mark on the scale above)

15b. In this patient, do you believe making this diagnosis of anxiety would be helpful for his or her continued management?

Not helpful at all — Very helpful

(Place a mark on the scale above)

15c. If the patient described above were < 25 years old, how comfortable would you be in making the diagnosis of anxiety?

Not comfortable at all — Very comfortable

(Place a mark on the scale above)

16. To what degree would each of the following increase your level of comfort in making a diagnosis of anxiety in patients with chest pain and providing a referral for treatment?

16a. Practice patterns of colleagues

Not helpful at all — Very helpful

(Place a mark on the scale above)

16b. Local hospital policy adoption

Not helpful at all — Very helpful

(Place a mark on the scale above)

16c. Multicenter trial

Not helpful at all — Very helpful

(Place a mark on the scale above)

16d. Professional organization practice guidelines

Not helpful at all — Very helpful

(Place a mark on the scale above)

17. What level of professional responsibility do you believe you have to provide patients with an actual diagnosis of anxiety when life threats are ruled out?

No responsibility at all — Very much responsibility

(Place a mark on the scale above)
