# Supplementary material for: Anxiety about anxiety: a survey of emergency department provider beliefs and practices regarding anxiety-associated low risk chest pain
Source: BMC Emerg Med. 2018 Mar 14;18:10. doi: 10.1186/s12873-018-0161-x (PMC5853064; doi:10.1186/s12873-018-0161-x)
Supplement: Supplementary file 2 — Table S1. Hypothetical Patient Scenario. Responses to questions regarding a hypothetical presentation of a patient with chest pain and suspected anxiety or panic. (DOC 224 kb) [file 12873_2018_161_MOESM2_ESM.doc]

**Supplemental Table 1: Hypothetical Patient Scenario**

| **Scenario:** In a patient with chest pain you suspect is driven by panic or anxiety, whom you plan to discharge home, with an age 50 years, a normal or unchanged ECG, a normal chest x-ray, and a normal troponin. | |
| --- | --- |
|  | mean (95% CI) |
| In this patient, how comfortable are you making the diagnosis of anxiety? | 51% (48-54) |
| In this patient, how helpful would making this diagnosis of anxiety be for his or her management? | 57% (55-60) |
| If the patient were <25 years, how comfortable would be making the diagnosis of anxiety? | 72% (70-75) |
